# External jugular vein aneurysm presenting as submandibular neck mass

**DOI:** 10.1093/jscr/rjac244

**Published:** 2022-05-31

**Authors:** Matthew I Saleem, Tristan Tham, Anise M Diaz, Patrick Keating, Alexandros Georgolios

**Affiliations:** 1 Department of Otolaryngology, Donald and Barbara Zucker School of Medicine at Hofstra/Northwell, New York, NY, USA; 2 Poplar Bluff Regional Medical Center, Poplar Bluff, MO, USA

## Abstract

External jugular vein aneurysms presenting as neck masses is very rare in the literature. This case report presents an 80-year-old female, who was referred to the office due to an incidental finding of a left submandibular neck mass. The patient presented to her primary care physician initially complaining of bilateral intermittent ear pain that was present for several years. After extensive workup, the patient was diagnosed with a left external jugular vein aneurysm. When asymptomatic, this rare condition can be followed safely on an outpatient basis. Vascular surgery consultation should also be considered.

## INTRODUCTION

Venous aneurysms located in the head and neck are reported to have an indolent clinical presentation frequently associated with local pain or tenderness without significant risk for severe complications such as embolism and rupture [1, 2]. The most common structure involved is the internal jugular vein, followed by the external jugular vein, and least frequently the anterior jugular vein [3]. To the best of our knowledge, this is the first presentation of an external jugular vein aneurysm masquerading as a submandibular neck mass.

## CASE REPORT

**Figure 1:**
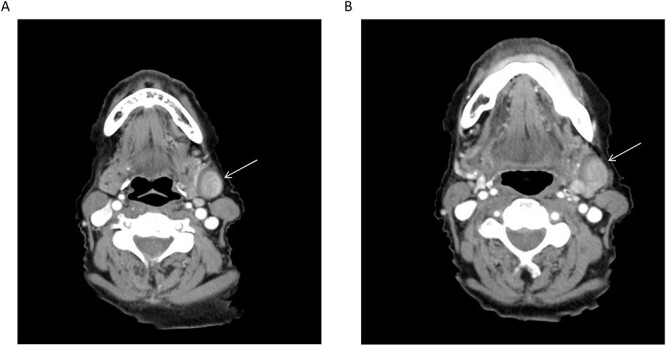
(**A**, **B**) Axial CT cuts of the neck with contrast revealing the lesion in the left submandibular neck (arrow); retrospective review demonstrates communication of the lesion with the adjacent left external jugular vein.

An 80-year-old female was referred to the otolaryngology clinic by her primary care provider with the chief complaint of bilateral intermittent otalgia. During the encounter, the patient recalled mild, left submandibular neck edema associated with tenderness that would resolve in 2–3 days. These symptoms were long-standing and were deteriorating several months prior to her presentation to our office. The patient presented with a computed tomography (CT) ordered by her primary care provider, which revealed a hyperdense mass within the left submandibular gland measuring 1.8 cm anterior–posterior, 1.4 cm transverse and 1.8 cm in height (Fig. 1). This finding raised concern for a primary neoplasm of the left submandibular gland and she was subsequently referred to our clinic.

**Figure 2:**
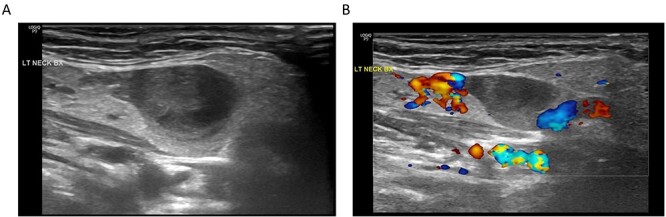
(**A**) Ultrasonography of the submandibular neck lesion; (**B**) ultrasonography of the lesion with color Doppler; the apparent lack of flow within the venous aneurysm is likely related to the imaging technique and relatively slow flow within the aneurysm compared to the adjacent external jugular vein.

The patient has a history of long-standing hypertension managed with enalapril and amlodipine. She also has hyperlipidemia which is managed with diet modification. On the day of the encounter, her vital signs were within normal range besides a mildly elevated blood pressure reading of 161/88 mmHg. Her physical examination and laryngoscopy were unremarkable, except for fullness on the left submandibular neck, without palpation of distinct masses or nodules. The discrepancy between physical examination and imaging prompted additional workup with an ultrasound (US) of the neck and request for fine-needle aspiration of the reported left submandibular neck mass. During the pre-intervention US, the vascular nature of the lesion was confirmed (Fig. 2). After real-time US evaluation by the interventional radiologist and further review of the prior neck CT, it was thought the lesion was most consistent with a venous aneurysm arising from the left external jugular vein at the level of the submandibular gland.

## DISCUSSION

The most common presentation for aneurysm in the neck region is a palpable soft mass that enhances with Valsalva maneuver [4, 5]. Doppler US is the gold standard and is recommended as first imaging technique to establish the diagnosis [6, 7]. Our patient presented from her primary care physician with a CT scan that raised suspicion for a neoplastic process and subsequent ultrasonography was required to establish the correct diagnosis. The classical classification for aneurysms includes primary (congenital) versus secondary (acquired) [8]. Known risk factors for the development of secondary venous aneurysms include recent trauma, cardiovascular disease and advanced age [9]. Our patient had a long-standing history of hypertension and it is unclear whether that was well controlled with her two daily per os medications, as her systolic blood pressure was elevated during the encounter.

There are rare reports of external jugular vein aneurysms in the literature [2, 10, 11], but the presentation of such an entity in the submandibular neck is exceedingly rare. In a case report of a patient with a facial vein aneurysm associated with acute submandibular gland sialadenitis, the authors hypothesized that inflammation of the gland had weakened the wall of the adjacent facial vein, causing aneurysmal dilatation [12]. Our patient had no strong evidence of acute or recurrent sialadenitis since she reported left submandibular neck pain episodes that were mild, intermittent, lasting for 2–3 days and without excessive local edema. The reported otalgia which prompted the initial work up by her primary care provider was bilateral, mild, intermittent and was attributed to temporomandibular joint myofascial pain syndrome.

The management of external jugular vein aneurysms can be observational, provided that these lesions are asymptomatic. Surgical intervention can be indicated either for cosmetic reasons when there is significant deformity or when complications arise due to phlebitis or thrombosis. Aneurysmal ruptures and pulmonary embolism are considered to be rare and surgical excision is thought to eliminate the risk of these severe complications [6]. Additionally, surgical management will provide a definitive histopathologic diagnosis of the lesion.

This case report illustrates the need to consider external jugular vein aneurysm in the differential diagnosis of upper neck masses. A meticulous head and neck physical examination, including Valsalva maneuver, should be performed and ultrasonography can confirm the diagnosis when suspicion is raised. When uncomplicated and asymptomatic, the lesion can be managed with watchful waiting. If intervention is required, appropriate preoperative planning will decrease the risk of intraoperative vascular complications.
